# Prevalence and Determinants of Overweight and Obesity Among Romanian Children Aged 5–17: A Cross-Sectional Study

**DOI:** 10.3390/jcm14103331

**Published:** 2025-05-10

**Authors:** Anca Cristina Drăgănescu, Alexandru Dinulescu, Daniela Păcurar, Viorel Jinga, Doina Anca Pleșca

**Affiliations:** 1 Faculty of Medicine, Department of Pediatrics and Department of Urology, “Carol Davila” University of Medicine and Pharmacy, 050474 Bucharest, Romania; anca.draganescu@umfcd.ro (A.C.D.); daniela.pacurar@umfcd.ro (D.P.); viorel.jinga@umfcd.ro (V.J.); doina.plesca@umfcd.ro (D.A.P.); 2 “Matei Bals” National Institute of Infectious Diseases, 021105 Bucharest, Romania; 3 Emergency Hospital for Children “Grigore Alexandrescu”, 011743 Bucharest, Romania; 4 “Prof. Dr. Theodor Burghele” Hospital, 050653 Bucharest, Romania; 5 Academy of Romanian Scientists, 050085 Bucharest, Romania; 6 Children’s Clinical Hospital Dr. Victor Gomoiu, 022102 Bucharest, Romania

**Keywords:** obesity, overweight, excess of weight, fast food, childhood obesity, obesity risk factors, obesity protective factors

## Abstract

**Background/Objectives**: Overweight and obesity in children is a worldwide health concern, with a high prevalence and an increasing trend in recent years. The prevalence of pediatric overweight and obesity in Romania is unclear; some studies estimate the rate of overweight at 15–20% and the obesity rate at 8.7–10.7%. The objective of this study is to measure the prevalence of overweight and obesity in children in Romania and to highlight their risks and protective factors. **Methods**: A repeated cross-sectional study that included children between 5 and 17 years old was performed across 2 years. Anamnestic data regarding demographics, family, and child’s lifestyle was collected from the child’s parent, and some medical and anthropometric parameters of the child were measured. The BMI and z-scores were calculated using the WHO charts. Logistic regression models were verified for goodness-of-fit and used for estimating the prediction value of fast-food consumption, parents’ education, and the presence of parents with obesity in the case of increased weight in the child. **Results**: A total of 1231 children were included in the study, 25.1% of whom exhibited an excess of weight. The prevalences of overweight and obesity were 17.5% and 7.6%, respectively. In the multivariate model, the following variables significantly predicted the weight of children: days of fast-food consumption, parents’ education, parents with excess weight, and hours of physical activity (*p* < 0.005) **Conclusions**: The prevalence of pediatric overweight and obesity in Romania is in accordance with the global prevalence. Considering fast-food consumption and the presence of parents with obesity as risk factors for children’s overweight or obesity and physical activity and a higher level of education of the parent as protector factors, we strongly emphasize the importance of public health initiatives directed towards these factors.

## 1. Introduction

The World Health Organization (WHO) defines overweight in children between 5–19 years old as a z-score greater than 1 standard deviation (SD) and obesity as a z-score greater than 2 SD for body mass index (BMI) [1]. Overweight and obesity in children is a worldwide health concern, with a high prevalence and an increasing trend in recent years. According to WHO data, prevalence tends to be higher in high-income countries (e.g., the USA and the UK), but increasing trends have also been observed in low- and middle-income countries (LMICs), including regions in Southeast Asia and Latin America [2,3,4]. The overall prevalence of overweight is estimated at around 14.8%, and the prevalence of obesity at around 8.5%, meaning that around 23.3% of children are either living with overweight or obesity, i.e., 1 in 4 children [2].

Overweight and obesity result from an imbalance between the intake and the expenditure of calories [5]. The intake of calories/day required for children varies with age and sex, ranging from 100 cal/kg/day in infants to 1800–2200 kcal/day for 12-year-olds [6]. Factors that contribute to achieving this status include genetic susceptibility, environmental factors, and behavioral factors. Genetic obesity occurs in less than 5% of cases in the pediatric population, but genetic susceptibility may play a role in all cases of children living with obesity, along with many other factors, such as the diet of the child in early life; therefore, the genetic factor is not the cause of the increased rate of childhood overweight and obesity. The factors that play a more significant role in the dramatic increase in childhood overweight and obesity in recent decades could be fast-food and sugary beverage consumption, large portion sizes of the meals, sedentary lifestyle, extensive usage of electronic devices, and family habits [7,8,9,10]. An important aspect that contributes to the high consumption of fast-food and sugary beverages is the natural preference for sweet and salty tastes in children [11,12]. A new factor (and it should not be neglected) contributing to childhood weight gain over the last few years is the COVID 19 pandemic [13,14,15].

In the long-term, obesity has the potential to affect all body systems. Hypertension is strongly associated with childhood obesity, being estimated be present in about ¼ of children with obesity. Type 2 diabetes mellitus (DM) is also closely linked with childhood obesity; around 85% of the children with type 2 DM are living with overweight or obesity. Dyslipidemia is also a common complication of pediatric obesity. The complications of obesity are increased risks for the development of cardiovascular disease (CVD), strokes, and kidney failure [16,17,18,19]. Other complications include pulmonary-related issues (asthma and sleep apnea) or mineral deficiencies [20,21]. The psychosocial impact of obesity, e.g., body dissatisfaction, depressive disorder, anxiety disorder, and even suicidal thoughts, must not be overlooked [22,23].

**Figure 1:**
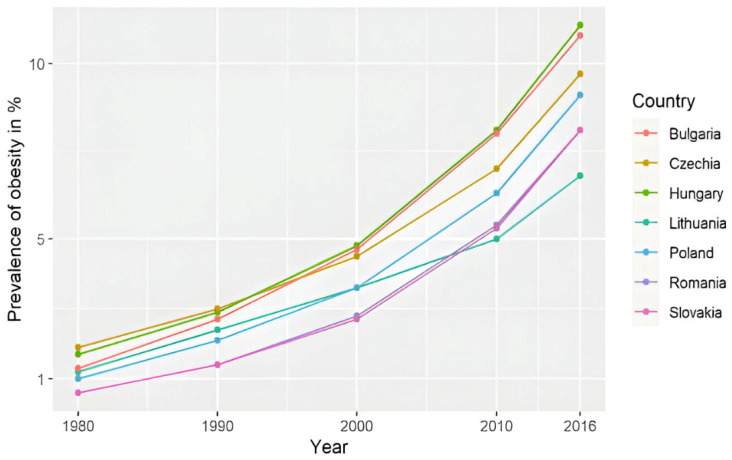
The prevalence of obesity in children and adolescents in countries from Central and Eastern Europe, including Romania [25].

The reported prevalence of overweight and obesity in the adult population of Romania is very high, with around 30% of the adult population being overweight and 21–26% living with obesity [24,25,26,27]. The prevalence of pediatric overweight and obesity in Romania is unclear; some studies estimate the overweight rate at 15–20% and the obesity rate at 8.7–10.7%, with a constantly increasing prevalence from 1980 to 2016 (Figure 1) [28,29,30,31]. The WHO Childhood Obesity Surveillance Initiative (COSI), round 6 (2022–2024), estimates that 28% of 7–9 years old children from Romania are living with weight excess (overweight and obesity), and 12% of these children live with obesity. Between round 5 (2020–2022) and round 6, the prevalence was decreasing in girls of this age (−0.7%), but what is more alarming is the increase of 2.9% in boys, with Romania, in this report, being the second country (after Austria-3%) in terms of the rate of increase in this prevalence in boys in this age group (Brief review of results from round 6 of COSI [32].

The objective of this study is to measure the current prevalence of overweight and obesity of children in Romania, their prevalence by the country regions, and to study the factors that may positively/negatively influence the weight of children in this country.

## 2. Materials and Methods

This is a repeated cross-sectional study performed by a team of pediatricians across 2 years, in the summers of 2023 and 2024, over a period of 5 days each summer in health centers in Romanian holiday resorts during the holiday period, including respondents from all over the country.

The children, aged 5–17 years old, participated voluntary in the study. All the parents signed an informed consent form, approving the inclusion of their child in the study.

Anamnestic data were collected from the child’s parent, and some medical and anthropometric parameters (weight and height) were measured by the doctors for each child. The BMI and z-scores were calculated using the WHO charts, using standard deviations (SD). The anamnesis consisted of demographics questions, i.e., sex, age, and provenance, family data; the International Standard Classification of Education (ISCED) of the parents and the existence of a parent/sibling with excess weight; and the child’s lifestyle (the existence of a meal schedule, how many times a week the child consumed fast food, how many hours a week the child practiced sports, and how many hours a day the child sat in front of electronic screens).

The margin of error for a confidence interval (CI) of 95% was 3%, calculated by the following formula: ε = z × σ/√n.

ε = margin of error;
z = z-score; 1.96 for a CI of 95%;
σ = population standard deviation; 4 million children;
n = sample size; 1231 respondents.

The z-score of BMI was calculated using the Ped(z) pediatric calculator and was interpreted using WHO references (Table 1) [1].

In the text, excess weight was defined as overweight or children living with obesity.

The International Standard Classification of Education (ISCED), adapted to our country, was used to classify the education of the parents (Table 2). We noted the highest education level in the family (father or mother) [33].

The numbers of respondents were counted for each county of Romania, i.e., 41 counties and the capital city of Bucharest. In addition, the individuals were according to one of the eight development regions of Romania:

1. North-East: Bacău, Botoșani, Iași, Neamț, Suceava, and Vaslui.
2. South-East: Brăila, Buzău, Constanța, Galați, Tulcea, and Vrancea.
3. South-Muntenia: Argeș, Călărași, Dâmbovița, Giurgiu, Ialomița, Prahova, and Teleorman.
4. South-West Oltenia: Dolj, Gorj, Mehedinți, Olt, and Vâlcea.
5. West: Arad, Caraș-Severin, Hunedoara, and Timiș.
6. North-West: Bihor, Bistrița-Năsăud, Cluj, Maramureș, Satu Mare, and Sălaj.
7. Center: Alba, Brașov, Covasna, Harghita, Mureș, and Sibiu.
8. Bucharest–Ilfov: Bucharest and Ilfov [34].

The data were analyzed using IBM SPSS Statistics, version 25, and illustrated using Microsoft Office Excel/Word 2013. Quantitative variables were tested for normal distribution using the Shapiro–Wilk test and were used as medians with interquartile ranges (IQR). Quantitative variables were tested between two independent groups using Mann–Whitney U tests. The Kruskal–Wallis test was used to determine significant differences between three or more groups of independent variables. Fisher’s exact test was used to determine the nonrandom associations between categorical variables, with the Bonferroni method used for correction. Fisher’s exact test was used independently to assess the association between child overweight/obesity and several categorical risk factors, including parental weight status, sibling weight status, and the presence of a meal schedule. Although each test was performed separately, they addressed related hypotheses concerning predictors of excess weight. Therefore, Bonferroni correction was applied for multiple comparisons (n = 3), setting the adjusted significance level at α = 0.017. Fisher’s exact test was used to assess the association between nutritional status categories (severe thinness, thinness, normal weight, overweight, and obesity) and categorical variables such as regular fast-food consumption and area of provenance. A statistically significant association was found between regular fast-food consumption and nutritional status (*p* < 0.001), with a higher proportion of excess weight observed among children who reported regular fast-food intake. In contrast, no significant association was observed between area of provenance and nutritional status (*p* = 0.987). As these comparisons were conducted independently and addressed distinct hypotheses, correction for multiple testing was not applied in this context. Logistic regression models were verified for goodness-of-fit and used for estimating the prediction value of fast-food consumption, parents’ education, physical activity, screen time, regular meal schedule, and the presence of a parent/sibling with obesity in case of increased children weight (in univariate and multivariate models). The regression used continuous variables, i.e., fast-food consumption (how many times per week), physical activity (hours/week), parents’ education (ISCED score), and screen time (hours/day), and categorical variables that were defined as yes/no, i.e., excess weight in parents/siblings (BMI over 25 kg/m^2^) and meal schedule (the child had the practice of eating at designated times throughout the day more than 5 days/week).

In our logistic regression analysis, the model fit was evaluated using the Hosmer–Lemeshow test, which indicated that the model sufficiently fit the data (*p* = 0.44). For multicollinearity testing, variance inflation factor (VIF) and tolerance were measured, and both were close to one for each variable; therefore, multicollinearity does not exist in the regression model.

## 3. Results

In the period of the study, 1439 voluntary respondents under 18 years old were included. A total of 1231 children were included in the cohort (194 children did not meet the age criteria as aged 5–17 years, and there were missing data for another 14 children). A total of 621 (50.4%) were female, and 610 (49.6%) were male (Table 3).

**Figure 2:**
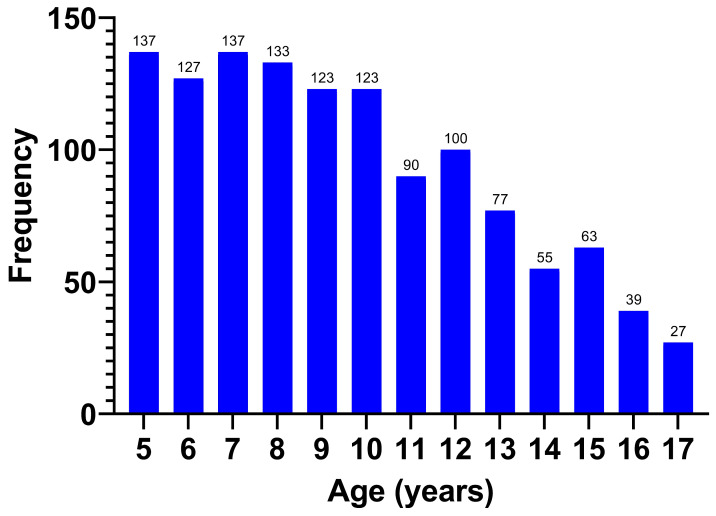
Age of the respondents in years.

The age of the respondents displayed a non-normal distribution (*p* < 0.001), with a median of 9.25 (7–12) years (Figure 2).

The respondents were classified by age group (Table 3), with the majority coming from the 5–7 years age group (32.6%).

After calculating and interpreting the BMI and the z-scores, we classified the weight of the children (Table 3). The BMI z-score showed a normal distribution (*p* = 0.062), with an average of +0.11 ± 1.29 SD

A total of 364 (29.6%) children were not classified as normal weight for their age, with most of them being overweight. Almost a quarter of all the participants, 310 (25.1%) children, were either living with overweight or with obesity, and 54 (4.5%) were classified as exhibiting thinness or severe thinness. The rate of obesity was 7.6%, and of that of overweight was 17.5%. The mean z-score of the overweight children was +1.44 ± 0.27 SD.

Weight excess affected 24.5% of females and 25.9% of males, with similar obesity rates (7.4% vs. 7.9%) and no significant gender difference (*p* = 0.584).

We found no correlation between age and the weight class (*p* = 0.59) or the age group and the weight class (*p* = 0.296).

The majority came from urban areas, 937 (76.1%). We found no association between area of provenance and the weight class (*p* = 0.987). This can be explained by the fact that the rural/urban division no longer respects the degree of development, as there are rural environments, such as those around the capital city or other large cities, which are more developed than some urban environments in the country.

**Figure 3:**
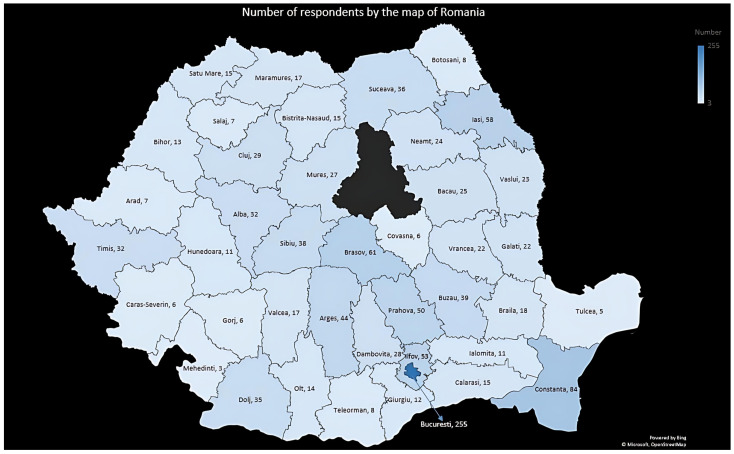
Number of respondents shown on a map of Romania (by county).

The study included respondents from 40 of the 41 counties of Romania and the capital city of Bucharest; most of them, 255 (20.7%), were from Bucharest, (Figure 3). Dividing the respondents by region, the highest prevalence of excess weight was found in the South-West Oltenia region (37.3%) and the lowest in the North-East region (19.6%), but it should be noted that the number of respondents by region was not equally distributed (Table 4).

Most of the children came from a high education background, 763 (62%), followed by medium education, 418 (34%), and the fewest came from a low education background, 50 (4%). The rate of excess weight was higher in those with a medium education background than in those with parents of high education (30.9% vs. 21.5%) **(*p* = 0.001)**, and the ISCED was higher in the group with no excess weight (6 (3–6) vs. 5 (3–6); ***p* < 0.001**).

**Figure 4:**
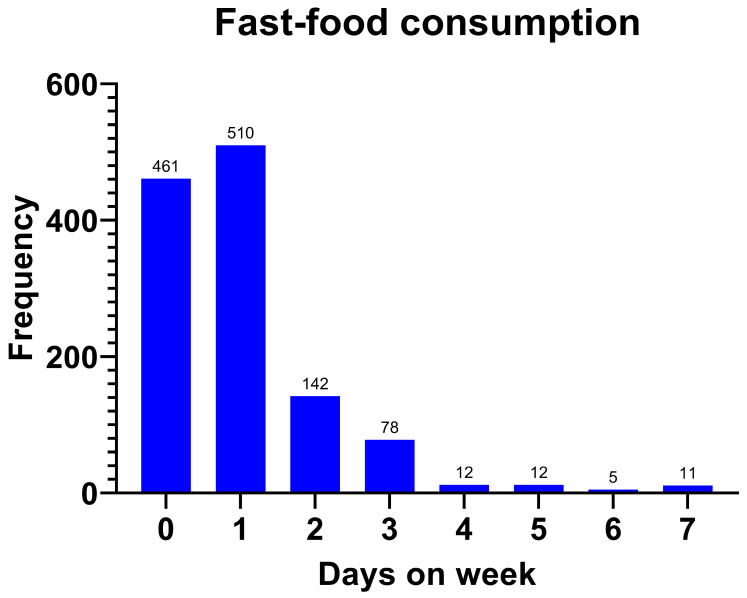
Fast-food consumption (days/week).

The majority families, 815 (66.2%), responded that they ate fast food. Of those responders who ate fast food, more than a half of the children, 510 (66.2%), confirmed that they consumed this type of food only once a week (Figure 4).

The rate of fast-food consumption was higher in the children with excess weight than in normal the weight/thinness group (77.4% vs. 57.5%) **(*p* < 0.001)**. The fast-food consumption rate by individual groups was 75.5% reporting regular consumption in the obesity group and 78.2% reporting regular consumption in the overweight group (*p* < 0.001; Table 5), and the group with excess weight consumed fast food more frequently (1 (0–1) vs. 1 (1–2) days/week; ***p* < 0.001**).

A total of 795 (64.6%) children responded that they engaged in physical activity, with a median of 4 (2–6) hours/week. The rate of excess weight was higher in those who did not report engaging in physical activity (29.4%) than in those without regular physical activity (22.9%) (*p* = 0.013). The number of hours/week of physical activity was lower in the excess weight group, 2 (0–4) hours, compared with that reported by the other group, 2 (0–5) hours (*p* = 0.02).

A total of 400 (32.5%) had at least one parent with excess weight, 309 (25.1%) had one excess weight parent, and 91 (7.4%) possessed both parents with excess weight.

The proportion of children with excess weight is significantly higher in the group with at least one parent with excess weight (34.3%) than in the group with neither parent with excess weight (20.8%) (*p* < 0.001).

A total of 893 (72.5%) of the children included in the study had siblings; 92 (7.2%) of those had at least a sibling with excess weight, and the rate of excess weight was higher in those with an excess weight sibling (38% vs. 22.7%) (*p* = 0.004).

The majority, 908 (73.8%), of families responded that they had a meal schedule. The children with a meal schedule had a lower excess weight rate (22.2%) than those without a definite meal schedule (33.4%) (*p* < 0.001).

They were asked how much screen time the children were allowed in 1 day, with answers ranging from 1 to 8 h. Almost half of them (42.9%) responded that they watched electronic devices for a maximum of 2 h/day. The average screen time was 3.25 ± 1.85 h/day. Children with excess weight had significantly higher screen time (median: 5 vs. 4 h/day; *p* = 0.001).

Significant associations were observed between excess weight and the following variables: fast-food consumption, ISCED of the parents, the presence of at least one parent with excess weight, siblings with excess weight, physical activity, and a meal schedule, variables that were further used in the regression (summary in Table 6).

Data from Table 7 represents logistic regression models for the overweight/obesity status of the children. In univariate models, all included variables significantly predicted the weight of the children.

A multivariate regression model was created using the variables included in the univariate models, i.e., the days of fast-food consumption, parents’ ISCED, parents with excess weight, siblings with excess weight, hours of physical activity/week, the existence of a meal schedule, and screen time/day as prediction variables. In this model, fast-food consumption, parents’ ISCED, the existence of an excess weight parent, and physical activity had a significant effect (*p* < 0.005).

The fact that meal schedule did not have a significant effect in the multivariable regression can be explained by the fact that the existence of a regular/irregular meal schedule is predictable according to the frequency of days of fast-food consumption (0.75; 95% CI; 0.68–0.83; *p* < 0.001), the predictive effect of the meal schedule decreasing in the multivariable logistic regression.

The information that the existence of siblings with excess weight did not have a significant effect in the multivariable regression can be explained by the fact that the existence of a sibling with excess weight is predictable according to the existence of a parent with obesity (3.63; 95% CI; 2.3–5.73; *p* < 0.001) and the ISCED of the parents (0.78; 95% CI; 0.68–0.9; *p* = 0.001), the predictive effect of the siblings decreasing in the multivariable logistic regression.

## 4. Discussion

### 4.1. Global Context

At the global level, the prevalence of childhood excess weight differs according to different criteria, such as the socioeconomic levels of the countries, the age group, or the sex. In the United States of America (USA), the prevalence of obesity among 2–18-year-olds was almost 1 in 5 children (19.7%), not including those who were overweight [35].

In the European context the highest prevalence of excess weight in children was reported in the United Kingdom (UK), at a rate of 29%, followed by Italy, with 26%. The lowest rates were reported in Slovakia (9%), followed by Turkey and The Netherlands, both with 10.5% [29]. If we compare these rates with those from this study, we can conclude that Romania has one of the highest rates of excess weight among children in Europe.

In Romania and its neighboring countries, the prevalence of excess weight among children ranges from 19.7% in Ukraine to 34.8% in Serbia, with Romania (23.7–30.7%) falling in the mid-range, comparable to the levels for Bulgaria (30.2%) and Hungary (20%) [28,29,30,36,37,38,39].

Almost ¼ (25.1%) of the children included in this study had an excess of weight. The prevalences of overweight and obesity in our study were 17.5% and 7.6%, respectively. The prevalences we found were in agreement with the overall estimated prevalence, 14.8% for overweight and 8.5% for obesity, and the prevalences found in this study are similar to those reported for the intervals previously described in Romania [2,28,29,30].

### 4.2. Risk Factors

The prevalence of overweight and obesity were slightly higher in boys than in girls, 18% vs. 17.1% and 7.9% vs. 7.4%, respectively, but with no statistical significance. The higher rate in male children is in concordance with the scientific literature in which a higher prevalence of excess weight in male children was reported [40,41,42]. In this study, there is no correlation between age or age group and the weight class, even though Raufil and Konstantiova (2022) reported an increase by age group in the prevalence of excess weight [40].

Parental influence emerged as a significant risk factor in our study, with the presence of excess weight in at least one parent increasing the child’s risk by 1.71 times. This association is well documented across multiple studies which consistently highlight the strong correlation between parental and child weight status [43,44,45,46,47,48]. A systematic review by Danford et al. (2014), which included 19 publications, emphasized the dual role of parents as both contributors to and potential agents of change in regards to childhood obesity. Interestingly, parental feeding practices—particularly those involving food restriction—were found to be inversely associated with a child’s weight status [49].

As expected, fast-food consumption was identified in our study as a significant risk factor for excess weight among children—a relationship that is well established in the literature [50,51,52,53,54]. Across multiple studies, a consistent positive association has been found between frequent fast-food intake and childhood obesity. Setiyaningrum and Rahmawaty (2023), in a review of six studies, reported that fast-food consumption is positively correlated with obesity in elementary school children [55]. Supporting this, Jakobsen et al. conducted a meta-analysis of 60 studies and found that high consumption of fast food increased the risk of excess weight by 1.17 times, while the consumption of sugar-sweetened beverages was associated with a 1.20 times higher risk (*p* < 0.05) [50]. Furthermore, a meta-analysis by Jiang et al. (2023) revealed that the prevalence of overweight and obesity among children is directly proportional to the density of fast-food outlets in their area. Even the presence of a single fast-food restaurant increased the risk of excess weight (OR = 1.033 [1.001, 1.066]), while greater distance to the nearest outlet was associated with a reduced risk (OR = 0.992 [0.988, 0.996], *p* ≤ 0.01) [56]. Collectively, these findings underscore the global public health burden posed by fast-food consumption and emphasize the urgent need for measures aimed at limiting children’s access to fast food.

The relationship between screen time and increased BMI in children remains a subject of debate, with mixed findings in the literature. While some studies suggest a positive association, others report no significant impact when screen time is addressed in isolation. A meta-analysis by Haghjoo et al. (2022), which included 44 studies, found that high screen time exposure was associated with a 27% increased risk of excess weight in children (OR = 1.273; 95% CI = 1.166–1.390; *p* < 0.001) [57]. Similarly, a cross-sectional study by Chang et al. (2023) observed that prolonged screen time in children aged 2–6 years increased the risk of obesity compared to that observed for moderate use, with each additional hour raising the risk by 10% [58]. However, a meta-analysis by Zhang et al. (2022), focusing on screen time interventions, found no significant reduction in obesity risk from reducing screen time alone, suggesting that screen exposure may need to be addressed alongside other lifestyle factors to be effective [59]. In our own study, while screen time was initially associated with excess weight, this relationship did not remain statistically significant in the multivariable model—possibly due to measurement limitations, as screen time was self-reported rather than objectively tracked.

### 4.3. Protective Factors

Our findings suggest that a higher parental educational level is associated with a healthier nutritional status in children—an observation supported by previous research. However, this relationship appears to vary by country development level. For instance, a 12-country study by Muthuri et al. (2016) reported that in high-income countries, such as the USA, increased parental education was linked to a lower risk of childhood overweight, whereas in low-income countries, like Kenya, higher maternal education was paradoxically associated with a greater risk of childhood overweight [60,61,62]. Given that Romania is classified as a developed country, our results align with patterns observed in other similar settings.

Sedentary behavior also emerged as a risk factor for excess weight in our cohort, consistent with the findings of the broader literature. For example, Liu et al. (2022) found a 45% increased risk of overweight/obesity in adolescents with sedentary behavior, and Mitchell et al. (2009) reported similar findings in a large cohort of over 5000 children [63,64]. These results, along with others, reinforce the role of physical activity as a protective factor against childhood overweight [63,65,66,67,68].

Family meal patterns have also been highlighted as influential in regards to weight outcomes. Several studies have shown that regular family meals can reduce the risk of overweight in children [69,70,71]. A recent large-scale study by López-Gil et al. (2024) demonstrated that children who ate daily family meals displayed the lowest predicted probabilities of overweight/obesity, and Mahmood et al. (2023) reported significantly lower odds of excess weight in children who ate breakfast or dinner with their families at least three times per week [69,70]. In our study, a structured meal schedule was associated with lower rates of excess weight; however, this association lost significance in the multivariable model, likely due to its overlap with the frequency of fast-food consumption, which appears to be a stronger predictor in this context.

### 4.4. Future Directions

Public health measures are needed to reduce the effect of risk factors and increase the effect of the protective factors described in this study. In order to act on the risk factors, it is very important to introduce campaigns to educate the population on the consumption of unhealthy foods through advertisements on television or, recently more importantly, through social media. These campaigns must also emphasize the importance of the family meal program. A way to improve the protective factors, such as including a more intensive practice of physical exercise, could be to promote local children’s teams for different sports.

Some countries have already introduced national programs to reduce excess weight in children; for example, in 2016, the UK government introduced a 10-year plan to reduce excess weight in children in their country. This plan includes a tax on the soft drink industry, a 20%, sugar reduction in children’s products, the promotion of physical education in schools, and supporting healthier school food [72].

In addition to national programs, excess weight must also be combated at the local level. Intervention programs look promising; for example, Farris et al. (2011) report a successful 12-week intervention program in children with obesity that consisted of exercise groups and nutrition education [73]. Some similar programs can be initiated by local governments in local school gyms.

In the future, there is a need for longitudinal studies to establish causal relationships or the impact of community interventions.

### 4.5. Limitations:

Methodological limitations include the reliance on self-reported data. In many cases, participants reported their own weight status and that of family members, rather than being directly measured by the authors. This introduces potential inaccuracies due to recall bias or social desirability bias. Furthermore, information regarding screen time, fast-food consumption, sedentary behavior, and meal scheduling was also self-reported, without objective validation, which may affect the reliability of these variables. Waist circumference-to-height ratio was not measured, even though this may be a better predictor of obesity [74].

Sampling limitations are also present. As participation in the study was voluntary, there is a risk of selection bias. Individuals with overweight or obesity might have been less likely to participate due to stigma or discomfort discussing weight-related issues. Additionally, the sample may underrepresent individuals from rural areas, further limiting generalizability.

Recommendations for future research include the use of objective measures where possible; for example, using wearable devices to track screen time and physical activity, or in-person weight measurements to validate reported data. To improve sample representativeness, stratified or randomized sampling techniques could be employed, and efforts should be made to actively engage underrepresented populations, particularly from rural communities.

## 5. Conclusions

The prevalence of pediatric excess weight in Romania aligns with global trends. This study identified fast-food consumption and parental overweight as significant risk factors, while higher parental education and increased physical activity emerged as protective factors. Although no significant association was found between screen time and excess weight, future studies should consider using objective measurements for more accurate assessment.

These findings underscore the importance of targeted public health strategies. Interventions should focus on promoting physical activity, reducing fast-food intake, and supporting parental education about healthy lifestyles. Empowering parents through education may be the most impactful approach, as they play a central role in shaping their children’s health behaviors. 

## Figures and Tables

**Table 1:** BMI z-score interpretation (WHO).

Z-Score	Weight
<−3 SD	Severe thinness
<−2 SD	Thinness
[−2–+1] SD	Normal weight
>+1 SD	Overweight
≥+2 SD	Obesity

**Table 2:** International Standard Classification of Education (ISCED), adapted to the Romanian educational system.

Education Level	ISCED	Score
Low education	Early childhood education (“less than primary” for educational attainment)	0
	Primary education	1
	Lower secondary education	2
Medium education	Upper secondary education	3
	Post-secondary non-tertiary education	4
High education	Short-cycle tertiary education	5
	Bachelor’s or equivalent level	6
	Master’s or equivalent level	7
	Doctoral or equivalent level	8

**Table 3:** Descriptive statistics of the main characteristics of the population.

Variable	N (%)	Median (IQR)
Gender		
Male	610 (49.6%)	
Female	621 (50.4%)	
Age (years)		9.25 (7–12)
5–7 years old	401 (32.6%)	
8–10 years old	379 (30.8%)	
11–13 years old	267 (21.7%)	
Over 13 years old	184 (14.9%)	
Weight class		
Normal weight	867 (70.4%)	
Overweight	216 (17.5%)	
Obesity	94 (7.6%)	
Thinness	43 (3.5%)	
Severe thinness	11 (0.9%)	
Excess weight	310 (25.1%)	
(Overweight + Obesity)		
Area of provenance		
Urban	937 (76.1%)	
Rural	294 (23.9%)	
Parents’ education (ISCED)		6 (3–6)
High education	763 (62%)	
Medium education	418 (34%)	
Low education	50 (4%)	
Physical activity		2 (0–4)
(hours/week)		
Fast-food consumption		1 (0–1)
(days/week)		
Screen time		3 (2–4)
(hours/day)		
Meal schedule		
Yes	908 (73.8%)	
No	323 (26.2%)	
Parents with excess weight		
Yes	400 (32.5%)	
No	831 (67.5%)	
Siblings with excess weight		
Yes	92 (10.3%)	
No	801 (89.7%)	

**Table 4:** Excess weight prevalence by Romania regions.

Region	Respondents (n)	Overweight (%)	Obesity (%)	Excess of Weight (%)
South-West Oltenia	75	24	13.3	37.3
South-East	190	22.6	9.5	32.1
North-West	96	22.9	8.3	31.2
South-Muntenia	168	19.6	6	25.6
West	56	16.1	8.9	25
Center	164	16.5	6.7	23.2
Bucharest–Ilfov	308	14	6.2	20.2
North-East	174	12.1	7.5	19.6

**Table 5:** Fast-food consumption rate by weight group.

Regular Fast-Food Consumption	Severe Thinness	Thinness	Normal Weight	Overweight	Obesity	Fisher’s Exact Test (*p*)
Yes	7 (63.6%)	18 (41.9%)	**498 (57.4%)**	**169 (78.2%)**	**71 (75.5%)**	**<0.001**
No	4 (36.4%)	25 (58.1%)	**369 (42.6%)**	**47 (21.8%)**	**23 (24.5%)**	

Note: The bold values indicate statistical significance.

**Table 6:** Association between excess weight in children and possible risk factors (fast-food consumption, ISCED of the parents, the presence of at least one parent with excess weight, physical activity, and a meal schedule).

Children with Excess Weight	Association
Days of fast-food consumption	*p* < 0.001 **
Parents’ ISCED	*p* < 0.001 **
Parents with excess weight	*p* < 0.001 *
Physical activity	*p* = 0.002 *
Siblings with excess weight	*p* = 0.004 *
Meal schedule	*p* < 0.001 *
Screen time	*p* = 0.001 *

* Fisher’s exact test; ** Mann–Whitney U test.

**Table 7:** Logistic regression for children with excess weight.

Parameter	Univariable		Multivariable	
	OR (95% CI)	*p*	OR (95% CI)	*p*
Fast-food consumption (days/week)	1.355 (1.223–1.502)	**<0.001**	1.317 (1.161–1.494)	**<0.001**
Parents’ ISCED	0.819 (0.758–0.886)	**<0.001**	0.867 (0.787–0.956)	**0.004**
Parents with excess weight	1.981 (1.519–2.585)	**<0.001**	1.714 (1.226–2.397)	**0.002**
Siblings with excess weight	2.088 (1.329–3.282)	**0.001**	1.441 (0.888–2.338)	0.139
Hours of physical activity/week	0.939 (0.904–0.976)	**0.001**	0.944 (0.903–0.987)	**0.012**
Meal schedule	0.570 (0.431–0.753)	**<0.001**	0.827 (0.577–1.185)	0.301
Screen time/day	1.128 (1.054–1.207)	**0.001**	1.035 (0.948–1.130)	0.439

## Data Availability

Data are contained within the article.

## References

[1] World Health Organization (WHO) (2022). Obesity and Overweight.

[2] Zhang X., Liu J., Ni Y., Yi C., Fang Y., Ning Q., Shen B., Zhang K., Liu Y., Yang L. (2024). Global Prevalence of Overweight and Obesity in Children and Adolescents. JAMA Pediatr..

[3] Lister N.B., Baur L.A., Felix J.F., Hill A.J., Marcus C., Reinehr T., Summerbell C., Wabitsch M. (2023). Child and Adolescent Obesity. Nat. Rev. Dis. Prim..

[4] World Health Organization WHO Issues Guidance on Emerging Double Threat of Childhood Obesity and Undernutrition in Low-and Middle-Income Countries. https://www.who.int/news-room/detail/05-06-2013-who-issues-guidance-on-emerging-double-threat-of-childhood-obesity-and-undernutrition-in-low--and-middle-income-countries.

[5] Sahoo K., Sahoo B., Choudhury A., Sofi N., Kumar R., Bhadoria A. (2015). Childhood Obesity: Causes and Consequences. J. Fam. Med. Prim. Care.

[6] Faizan U., Rouster A.S. (2025). Nutrition and Hydration Requirements in Children and Adults.

[7] Xu S., Xue Y. (2016). Pediatric Obesity: Causes, Symptoms, Prevention and Treatment. Exp. Ther. Med..

[8] Jebeile H., Kelly A.S., O’Malley G., Baur L.A. (2022). Obesity in Children and Adolescents: Epidemiology, Causes, Assessment, and Management. Lancet Diabetes Endocrinol..

[9] Becheanu C.A., Tincu I.F., Smadeanu R.E., Lesanu G. (2018). Feeding Practices among Romanian Children in the First Year of Life. Hong Kong J. Paediatr..

[10] Constantin A.T., Streata I., Covăcescu M.S., Riza A.L., Roșca I., Delia C., Tudor L.M., Dorobanțu Ș., Dragoș A., Ristea D. (2023). Genetic Testing for Familial Hypercholesterolemia in a Pediatric Group: A Romanian Showcase. Diagnostics.

[11] Mennella J.A. (2014). Ontogeny of Taste Preferences: Basic Biology and Implications for Health. Am. J. Clin. Nutr..

[12] Mennella J.A., Bobowski N.K. (2015). The Sweetness and Bitterness of Childhood: Insights from Basic Research on Taste Preferences. Physiol. Behav..

[13] Ferentinou E., Koutelekos I., Pappa D., Manthou P., Dafogianni C. (2023). The Impact of the COVID-19 Pandemic on Childhood Obesity: A Review. Cureus.

[14] World Health Organization (WHO) New WHO/Europe Report Highlights a Direct Link Between COVID-19 and Increased Obesity in School-Aged Children. https://www.who.int/europe/news/item/01-05-2024-new-who-europe-report-highlights-a-direct-link-between-covid-19-and-increased-obesity-in-school-aged-children.

[15] Iacopetta D., Catalano A., Ceramella J., Pellegrino M., Marra M., Scali E., Sinicropi M., Aquaro S. (2024). The Ongoing Impact of COVID-19 on Pediatric Obesity. Pediatr. Rep..

[16] Constantin A.T., Delia C., Tudor L.M., Rosca I., Irimie A.D., Năstase L., Gherghina I. (2023). Dyslipidemia in Pediatric Patients: A Cross-Sectional Study. Medicina.

[17] Balasundaram P., Krishna S. Obesity Effects on Child Health. https://www.ncbi.nlm.nih.gov/books/NBK570613/.

[18] American Diabetes Association (2000). Type 2 Diabetes in Children and Adolescents. Diabetes Care.

[19] Vajravelu M.E., Tas E., Arslanian S. (2023). Pediatric Obesity: Complications and Current Day Management. Life.

[20] Guta O.M., Iaru O.E., Vlad R.M. (2019). Eat Less to Sleep More—Sleep-Related Disorders in Obese Children, a Healthcare Problem. Rom. Med. J..

[21] Vlad R., Istrate-Grigore O.-A., Pacurar D. (2025). Customizing Nutrients: Vitamin D and Iron Deficiencies in Overweight and Obese Children—Insights from a Romanian Study. Nutrients.

[22] Rankin J., Matthews L., Cobley S., Han A., Sanders R., Wiltshire H.D., Baker J.S. (2016). Psychological Consequences of Childhood Obesity: Psychiatric Comorbidity and Prevention. Adolesc. Health Med. Ther..

[23] Newson L., Sides N., Rashidi A. (2024). The Psychosocial Beliefs, Experiences and Expectations of Children Living with Obesity. Health Expect..

[24] Cinteza M., Pana B., Cochino E., Florescu M., Margulescu A., Florian A., Vinereanu D. (2007). Prevalence and Control of Cardiovascular Risk Factors in Romania Cardio-Zone National Study. Mædica A J. Clin. Med..

[25] World Health Organization (WHO) (2022). WHO European Regional Obesity Report. https://www.who.int/europe/publications/i/item/9789289057738.

[26] Roman G., Bala C., Createanu G., Graur M., Morosanu M., Amorim P., Pîrcalaboiu L., Radulian G., Timar R., Achimas Cadariu A. (2015). Obesity and Health-Related Lifestyle Factors in the General Population in Romania: A Cross Sectional Study. Acta Endocrinol..

[27] Gallus S., Lugo A., Murisic B., Bosetti C., Boffetta P., La Vecchia C. (2015). Overweight and Obesity in 16 European Countries. Eur. J. Nutr..

[28] Radulescu C.R., Pleşca D.A. (2020). Prevalence and Possible Complications of Pediatric Obesity in Romania: A Review of Recent Literature. Rom. J. Pediatr..

[29] Pop T.L., Maniu D., Rajka D., Lazea C., Cismaru G., Ştef A., Căinap S.S. (2021). Prevalence of Underweight, Overweight and Obesity in School-Aged Children in the Urban Area of the Northwestern Part of Romania. Int. J. Environ. Res. Public. Health.

[30] Chirita-Emandi A., Barbu C.G., Cinteza E.E., Chesaru B.I., Gafencu M., Mocanu V., Pascanu I.M., Tatar S.A., Balgradean M., Dobre M. (2016). Overweight and Underweight Prevalence Trends in Children from Romania—Pooled Analysis of Cross-Sectional Studies between 2006 and 2015. Obes. Facts.

[31] Nittari G., Scuri S., Gamo Sagaro G., Petrelli F., Grappasonni I. (2021). Epidemiology of Obesity in Children and Adolescents. Teamwork in Healthcare.

[32] WHO (2024). Brief Review of Results from Round 6 of COSI (WHO European Childhood Obesity Surveillance Initiative) 2022–2024.

[33] Eurostat International Standard Classification of Education (ISCED). https://ec.europa.eu/eurostat/statistics-explained/index.php?title=International_Standard_Classification_of_Education_(ISCED).

[34] Surd V., Kassai I., Giurgiu L. (2011). Romania Disparities in Regional Development. Procedia Soc. Behav. Sci..

[35] Hu K., Staiano A.E. (2022). Trends in Obesity Prevalence Among Children and Adolescents Aged 2 to 19 Years in the US From 2011 to 2020. JAMA Pediatr..

[36] Dereń K., Wyszyńska J., Nyankovskyy S., Nyankovska O., Yatsula M., Łuszczki E., Sobolewski M., Mazur A. (2021). Secular Trends of Underweight, Overweight, and Obesity in Children and Adolescents from Ukraine. Int. J. Environ. Res. Public. Health.

[37] Jakab A.E., Hidvégi E.V., Illyés M., Cziráki A., Bereczki C. (2018). Prevalence of Overweight and Obesity in Hungarian Children and Adolescents. Ann. Nutr. Metab..

[38] Rangelova L., Petrova S., Konstantinova M., Duleva V., Dimitrov P. (2014). Overweight and Obesity Prevalence in Bulgarian Schoolchildren: A Comparison between Two International Standards. Int. J. Biomed. Adv. Res..

[39] Marković L., Đorđić V., Trajković N., Božić P., Halaši S., Cvejić D., Ostojić S.M. (2021). Childhood Obesity in Serbia on the Rise. Children.

[40] Raufi A., Konstantinova M.K. (2022). Prevalence of Overweight and Obesity in Children: Variation in Different Ethnicities, Age, and Sex in North Macedonia. Prilozi.

[41] Shah B., Tombeau Cost K., Fuller A., Birken C.S., Anderson L.N. (2020). Sex and Gender Differences in Childhood Obesity: Contributing to the Research Agenda. BMJ Nutr. Prev. Health.

[42] The Annie E. (2023). Casey Foundation Children and Teens Overweight or Obese by Gender in United States.

[43] Mikkelsen M., Mikkelsen M., Wilsgaard T., Grimsgaard S., Jacobsen B.K., Hopstock L.A. (2024). The Intergenerational Transmission of Obesity from Parents to Offspring: Insights from The Tromsø Study 1994–2016. Nor. Tidsskr. Ernæring.

[44] Ling J., Gebremariam M. (2023). Embracing Parenting Role in Childhood Obesity. BMC Public Health.

[45] Wang Y., Min J., Khuri J., Li M. (2017). A Systematic Examination of the Association between Parental and Child Obesity across Countries. Adv. Nutr..

[46] Tzou I.L., Chu N.-F. (2012). Parental Influence on Childhood Obesity: A Review. Health.

[47] Fuemmeler B.F., Lovelady C.A., Zucker N.L., Østbye T. (2013). Parental Obesity Moderates the Relationship between Childhood Appetitive Traits and Weight. Obesity.

[48] Lee J.S., Jin M.H., Lee H.J. (2022). Global Relationship between Parent and Child Obesity: A Systematic Review and Meta-Analysis. Clin. Exp. Pediatr..

[49] Danford C.A., Schultz C., Marvicsin D. (2015). Parental Roles in the Development of Obesity in Children: Challenges and Opportunities. Res. Rep. Biol..

[50] Jakobsen D.D., Brader L., Bruun J.M. (2023). Association between Food, Beverages and Overweight/Obesity in Children and Adolescents—A Systematic Review and Meta-Analysis of Observational Studies. Nutrients.

[51] Jia P., Luo M., Li Y., Zheng J., Xiao Q., Luo J. (2021). Fast-food Restaurant, Unhealthy Eating, and Childhood Obesity: A Systematic Review and Meta-analysis. Obes. Rev..

[52] Almuhanna M.A., Alsaif M., Alsaadi M., Almajwal A. (2014). Fast Food Intake and Prevalence of Obesity in School Children in Riyadh City. Sudan. J. Paediatr..

[53] Wang C., Zhen Z., Zhao N., Zhao C. (2021). Associations between Fast-Food Restaurants Surrounding Kindergartens and Childhood Obesity: Evidence from China. Int. J. Environ. Res. Public. Health.

[54] Libuy N., Church D., Ploubidis G., Fitzsimons E. (2023). Fast Food Proximity and Weight Gain in Childhood and Adolescence: Evidence from Great Britain. Health Econ..

[55] Setiyaningrum H.Y., Rahmawaty S. Association Between Fast-Food Consumption and Obesity in Elementary Students: Review Article. Proceedings of the International Conference on Health and Well-Being (ICHWB 2022).

[56] Jiang J., Lau P.W.C., Li Y., Gao D., Chen L., Chen M., Ma Y., Ma T., Ma Q., Zhang Y. (2023). Association of Fast-food Restaurants with Overweight and Obesity in School-aged Children and Adolescents: A Systematic Review and Meta-analysis. Obes. Rev..

[57] Haghjoo P., Siri G., Soleimani E., Farhangi M.A., Alesaeidi S. (2022). Screen Time Increases Overweight and Obesity Risk among Adolescents: A Systematic Review and Dose-Response Meta-Analysis. BMC Prim. Care.

[58] Chang R.-Y., Chen T.-L., Yeh C.-C., Chen C.-H., Wang Q.-W., Toung T., Liao C.-C. (2023). Risk of Obesity Among Children Aged 2–6 Years Who Had Prolonged Screen Time in Taiwan: A Nationwide Cross-Sectional Study. Clin. Epidemiol..

[59] Zhang P., Tang X., Peng X., Hao G., Luo S., Liang X. (2022). Effect of Screen Time Intervention on Obesity among Children and Adolescent: A Meta-Analysis of Randomized Controlled Studies. Prev. Med..

[60] Ding S., Chen J., Dong B., Hu J. (2021). Association between Parental Socioeconomic Status and Offspring Overweight/Obesity from the China Family Panel Studies: A Longitudinal Survey. BMJ Open.

[61] Seum T., Meyrose A.-K., Rabel M., Schienkiewitz A., Ravens-Sieberer U. (2022). Pathways of Parental Education on Children’s and Adolescent’s Body Mass Index: The Mediating Roles of Behavioral and Psychological Factors. Front. Public Health.

[62] Muthuri S.K., Onywera V.O., Tremblay M.S., Broyles S.T., Chaput J.-P., Fogelholm M., Hu G., Kuriyan R., Kurpad A., Lambert E.V. (2016). Relationships between Parental Education and Overweight with Childhood Overweight and Physical Activity in 9–11 Year Old Children: Results from a 12-Country Study. PLoS ONE.

[63] Mitchell J.A., Mattocks C., Ness A.R., Leary S.D., Pate R.R., Dowda M., Blair S.N., Riddoch C. (2009). Sedentary Behavior and Obesity in a Large Cohort of Children. Obesity.

[64] Liu H., Bi C., Lin H., Ma W., Zhang J., Hu Y.-Y., Liu J.-Z. (2022). Compared with Dietary Behavior and Physical Activity Risk, Sedentary Behavior Risk Is an Important Factor in Overweight and Obesity: Evidence from a Study of Children and Adolescents Aged 13–18 Years in Xinjiang, China. BMC Pediatr..

[65] Bezerra T., Souza Filho A., Quirino N., Bandeira P., Cabral L., Reuter C., Martins C., Carvalho F. (2023). Physical Activity, Sedentary Behaviour and Cardiovascular Risk Factors in Overweight Low-Income Schoolchildren: A Complex System Perspective. Obesities.

[66] Wyszyńska J., Ring-Dimitriou S., Thivel D., Weghuber D., Hadjipanayis A., Grossman Z., Ross-Russell R., Dereń K., Mazur A. (2020). Physical Activity in the Prevention of Childhood Obesity: The Position of the European Childhood Obesity Group and the European Academy of Pediatrics. Front. Pediatr..

[67] Hills A.P., Andersen L.B., Byrne N.M. (2011). Physical Activity and Obesity in Children. Br. J. Sports Med..

[68] Kawalec A., Mozrzymas R., Domżol A., Zachurzok A., Szczepańska M., Noczyńska A., Zwolińska D. (2024). Physical Activity and Its Potential Determinants in Obese Children and Adolescents under Specialist Outpatient Care—A Pilot Cross-Sectional Study. Healthcare.

[69] Mahmood L., Gonzalez-Gil E.M., Makrilakis K., Liatis S., Schwarz P., Herrmann S., Willems R., Cardon G., Latomme J., Rurik I. (2023). Cross-sectional and Longitudinal Associations between Family Meals Frequency and Children’s Overweight/Obesity in Families at High Risk of Type 2 Diabetes: The Feel4Diabetes-Study. Pediatr. Obes..

[70] López-Gil J.F., Ezzatvar Y., Ojeda-Rodríguez A., Galan-Lopez P., Royo J.M.P., Gaya A.R., Agostinis-Sobrinho C., Martín-Calvo N. (2024). Is Family Meal Frequency Associated with Obesity in Children and Adolescents? A Cross-sectional Study Including 155 451 Participants from 43 Countries. Pediatr. Obes..

[71] Saltaouras G., Kyrkili A., Bathrellou E., Georgoulis M., Yannakoulia M., Bountziouka V., Smrke U., Dimitrakopoulos G., Kontogianni M.D. (2024). Associations between Meal Patterns and Risk of Overweight/Obesity in Children and Adolescents in Western Countries: A Systematic Review of Longitudinal Studies and Randomised Controlled Trials. Children.

[72] HM Government Childhood Obesity: A Plan for Action. https://www.gov.uk/government/publications/childhood-obesity-a-plan-for-action.

[73] Farris J.W., Taylor L., Williamson M., Robinson C. (2011). A 12-Week Interdisciplinary Intervention Program for Children Who are Obese. Cardiopulm. Phys. Ther. J..

[74] Agbaje A.O. (2024). Waist-Circumference-to-Height-Ratio Had Better Longitudinal Agreement with DEXA-Measured Fat Mass than BMI in 7237 Children. Pediatr. Res..

